# Prevalence of Taurodont Molars in a Selected Iranian Adult Population 

**DOI:** 10.22037/iej.v12i3.13905

**Published:** 2017

**Authors:** Davoud Jamshidi, Maryam Tofangchiha, Nasim Jafari Pozve, Mahdis Mohammadpour, Bijan Nouri, Kazem Hosseinzadeh

**Affiliations:** a *Department of Endodontics, Dental School, Qazvin University of Medical Science, Qazvin, Iran; *; b *Department of Oral and Maxillofacial Radiology, Dental School, Qazvin University of Medical Sciences, Qazvin, Iran; *; c * Department of Oral and Maxillofacial Radiology, Dental Implants Research Center, Isfahan University of Medical Sciences, Islamic Azad University, Khorasgan Branch, Isfahan, Iran; *; d *Department of Epidemiology and Biostatistics,* *Medical School, Kurdistan University of Medical Science, Kurdistan, Iran; *; e *Faculty of Nursing* *and* *Midwifery, Qazvin University of Medical Sciences, Qazvin, Iran*

**Keywords:** Molar, Panoramic, Prevalence, Radiography, Taurodontism

## Abstract

**Introduction:**

Taurodontism is an anomaly characterized by elongated crowns and consumedly apical location of the bifurcation area. This study aimed to determine the prevalence of taurodontism in molars based on digital panoramic radiographies in eight cities of Iran.

**Methods and Materials:**

This descriptive cross-sectional study was conducted on 2360 digital panoramic radiographs taken for different treatment purposes. Demographic information of patients was recorded and radiographs were evaluated for presence of taurodont molars. The prevalence rates were calculated and the data were analyzed using SPSS software version 18 via paired *t*-test, *chi* square test and ANOVA.

**Results:**

A total of 2360 panoramic radiographs (from 51.4% male and 48.6% female patients) were evaluated and the prevalence of taurodontism was reported 22.9% (22.6% in males and 23.3% in females) (*P*>0.05). Its prevalence was 51.67% in the right and 48.33% in the left quadrants (*P*>0.05), 34.1% in the mandible and 65.9% in the maxilla (*P*=0.000) and 79.52% in the second and 20.48% in the first molar (*P*=0.000). The prevalence of hypotaurodontism, mesotaurodontism and hypertaurodontism was 84.13%, 11.07% and 4.8%, respectively.

**Conclusion:**

The prevalence of taurodont molars was high in Iran and it was more common in the second molars and in the maxilla. Hypotaurodontism had the highest prevalence.

## Introduction

Taurodontism is a developmental dental anomaly characterized by bull-like teeth. Taurodont teeth have vertically enlarged pulp chambers and apically displaced root bifurcation [1, 2]. This trait is caused by the failure of the Hertwig's epithelial sheath to invaginate at the proper horizontal level [3]. Taurodont teeth have wide variations in size and shape of pulp chamber and show morphological variations in terms of apically positioned root canal orifices or presence of extra roots [4, 5]. 

For the diagnosis of a normal or cynodont tooth, a crown.body:root (CB:R) ratio of 1:1 is reasonable, as it has been suggested that the length of the roots of a normal molar is at least equal to the crown-body length [6]. CB:R ratio of slightly less than 1:1 was considered to be within normal limits [7]. According to Shaw [7], taurodontism occurs in varying degrees that may be classified in increasing order of severity as hypotaurodontism, mesotaurodontism and hypertaurodontism. An objective classification based on the CB:R ratio determines that CB:R ratios of the range 1.10-1.29 be classified as the hypotaurodont group, those in the range 1.30-2.00 as the mesotaurodont group, and those more than 2.00 as the hypertaurodont group [8].

Evidence shows that taurodontism may be associated with genetic polymorphisms and syndromes such as tricho-dento-osseous syndrome, ectodermal dysplasia, Down syndrome, Klinefelter syndrome, hypophosphatasia and amelogenesis imperfecta. The characteristics of taurodont teeth associated with syndromes are similar to non-syndromic cases [9]. Clinically, taurodont teeth have an apparently normal crown [10]. This anomaly more commonly occurs in molar teeth. In general, permanent teeth are more commonly affected than primary teeth and it occurs unilaterally or bilaterally. This anomaly is easily detected radiographically and enlarged pulp chamber in apico-occlusal direction on radiographs clearly suggests taurodontism. Moreover, absence of cervical constriction and presence of short roots are among the major indicators of this anomaly [9]. 

Root canal treatment of a taurodont tooth is challenging [11-13]. Taurodont teeth often have abnormal root canals in terms of shape and number and due to complex anatomy of the roots; complete filling of the root canal system is a challenge [14]. Moreover, extraction of a taurodont tooth may be problematic due to widening of the apical third of the tooth. Also, it should be noted that in hypertaurodont teeth (most severe form of taurodontism), vital pulpotomy may be a suitable alternative to pulpectomy [15]. Endodontic post placement is not indicated in taurodont teeth. Also, since taurodont teeth occupy a smaller space in the alveolar bone, they have less stability than a normal tooth and this must be taken into account for orthodontic and prosthodontic treatments [13]. On the other hand, risk of furcal involvement is lower in taurodont teeth compared to normal teeth in periodontal disease and thus, taurodont teeth with periodontal disease often have a better prognosis than normal teeth with periodontal disease [15]. These variations in treatment planning of taurodont teeth necessitate further research on the prevalence of this anomaly in different geographical areas and its correlation with gender, type of teeth and side of the jaw. 

Prevalence of taurodontism was reported to be 8.6% in Saudi Arabia [16]. This rate was reported to be 3.9% in Korean dental patients with a higher prevalence in the mandible [17]. 

The prevalence of taurodontism in south of Iran was reported to be 5.5% and a higher prevalence was seen among females with a higher frequency of taurodont mandibular second molars [18]. However, the prevalence of this anomaly was found to be 0.5% in a study conducted in the north of Iran [19]. A study carried out in India reported the prevalence of taurodontism to be 2.5% with a higher frequency in females [20]. 

Controversy exists on the role of gender in occurrence of taurodontism [4], and a nationwide comprehensive study on the prevalence of taurodontism is lacking in Iran. Considering the variability in the reported prevalence rates for taurodontism in different geographical locations and the fact that this anomaly may lead to future dental problems and complicate certain dental treatments, its accurate diagnosis and knowledge about its prevalence seem necessary. This study aimed to assess the prevalence of taurodont molars on digital panoramic radiographs of Iranian population from different cities.

## Materials and Methods

This descriptive cross-sectional study was conducted on digital panoramic radiographs of patients presenting to the oral and maxillofacial radiology clinics of eight cities in Iran namely Urmia, Karaj, Sari, Qazvin, Isfahan, Zahedan, Ahvaz and Yazd, in 2015. On the map, Iran was theoretically divided into a northern and a southern part and four cities were randomly selected from each of the northern and southern parts. 

Sample size was calculated to be 2360 considering the confidence interval of 95%, expected prevalence of 0.20 and error rate (d) of 0.015. Thus, 295 panoramic radiographs were reviewed in each city. Radiographs of patients were evaluated anonymously. 

A total of 2360 panoramic radiographs ordered for variable diagnostic purposes (third molar extraction surgery, orthodontic treatment, periodontal assessment, *etc.*) were evaluated. The exclusion criteria were presence of open apex molar teeth, congenital missing of posterior teeth, presence of systemic diseases or syndromes (with a visible impact on panoramic radiographs), extracted or endodontically treated posterior teeth, teeth with large restorations and low-quality of radiographs. Demographic information of patients was recorded. All radiographs were inspected by a trained post-graduate student of endodontics. Using a sample of 100 radiographs, the intra-observer agreement was calculated using Cohen’s kappa statistics (kappa=0.97). Digital panoramic radiographs were displayed on a computer monitor. The brightness and contrast of the monitor was adjusted as standard prior to viewing. Digital images were coded and measurements were made on digital radiographs using Scanora software version 5.0 (Soredex, Helsinki, Finland). The values were recorded in the respective forms. 

**Figure 1:**
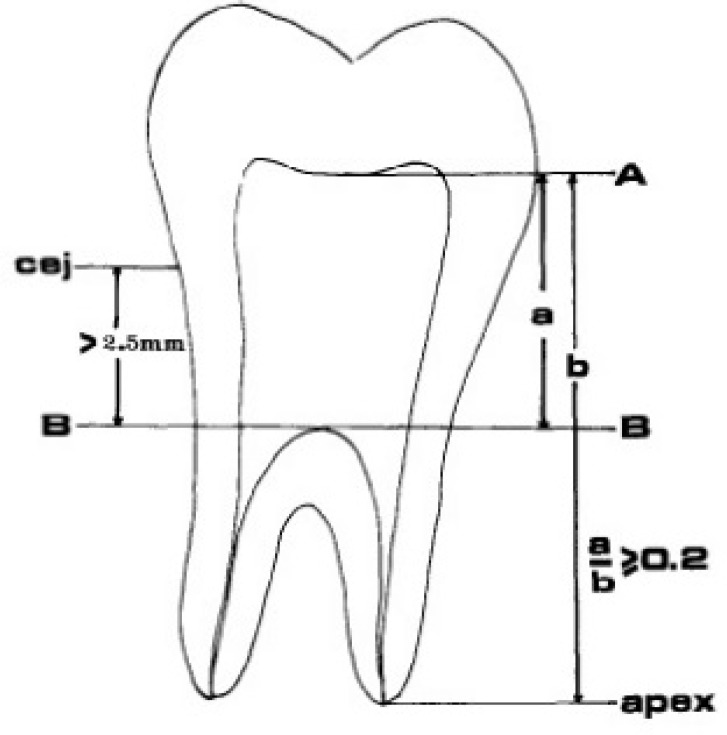
Detection of a taurodont tooth based on the criteria described by Shifman and Chanannel

The teeth were assessed based on the criteria described by Shifman and Chanannel [21]. The distance from the most apical point in the pulp chamber roof (A), to the most coronal point in the pulp chamber floor (B), was measured (a) and divided by the distance between the pulp chamber roof and apex of the longest root (b), and the obtained value was multiplied by 100. If the obtained value was ≥20 and the distance from the cementoenamel junction to pulp chamber floor (B) was more than 2.5 mm, the respective tooth was a taurodont tooth (Figure 1). Taurodont teeth were then classified based on their degree of severity as follows: hypotaurodontism; mild form, with the above-mentioned value between 20%-29.9%, mesotaurodontism; moderate form, with the above-mentioned value between 30%-39.9% and hypertaurodontism; severe form, with the above-mentioned value between 40%-75% [21].

All gathered data were coded and statistical analyses were carried out using SPSS version 18 (SPSS Inc., IL, USA). To determine the overall prevalence of taurodontism, the ratio of taurodont teeth to the total number of studied subjects was calculated and reported in percentages. The *chi* square test was used to compare the prevalence of taurodontism in the maxilla and mandible, and in the right and left quadrants. Also, *chi *square test was applied to assess the differences of severity of taurodontism with the jaw (maxilla/mandible) and quadrant (right/left). In addition, *chi* square test, was used to compare the prevalence of taurodontism among the eight cities.

## Results

A total of 2360 panoramic radiographs were evaluated; out of which, 51.6% belonged to males and 48.4% belonged to females. The overall prevalence of taurodontism was 8.84% of all molars examined and 22.9% of all patients (542 out of 2360 individuals); this rate was 22.6% in males and 23.3% in females. The association of taurodontism and gender was not statistically significant (*P*>0.05) (Table 1). 

**Figure 2:**
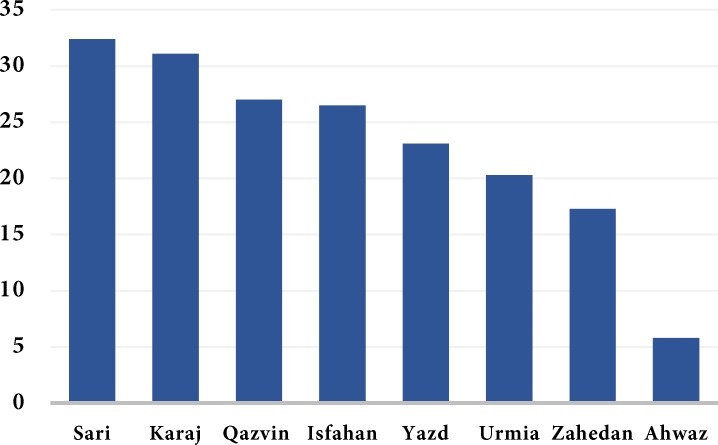
Prevalence rate of taurodontism in various Iranian cities

In general, 34.1% of taurodont teeth were in the mandible and 65.9% were in the maxilla; this difference was statistically significant (*P*=0.000). The prevalence of taurodontism was 48.33% in the left and 51.67% in the right quadrant which was not statistically significant (*P*>0.05). Prevalence of taurodontism was found to be 20.3% in Urmia, 32.4% in Sari, 27% in Qazvin, 31.1% in Karaj, 26.5% in Isfahan, 17.3% in Zahedan, 5.8% in Ahwaz and 23.1% in Yazd (Figure 2). The association between the prevalence of taurodontism and city was statistically significant (*P*=0.000). In other words, significant differences existed in prevalence of taurodont teeth among the cities, and Sari and Karaj had significantly the highest prevalence of taurodontism while its prevalence was significantly lower in Ahwaz and Zahedan. 

Bilateral taurodontism was found on 236 radiographs (8.1% of all patients); out of which, 32 were seen in the first molars and 204 were seen in the second molars. Of bilateral taurodont teeth, 167 cases were in the maxilla and 69 were in the mandible. The highest frequency of bilateral taurodontism belonged to the maxillary second molars and hypotaurodont group (Table 2). 

In terms of severity of taurodontism, 84.13% were hypotaurodont, 11.07% were mesotaurodont and 4.8% were hypertaurodont. The frequency of hypotaurodont, mesotaurodont and hypertaurodont teeth was significantly different among the eight cities (*P*=0.000), and generally, the frequency of hypotaurodontism was significantly higher than mesotaurodont teeth (*P*=0.000). A significant association was noted between type of taurodontism in terms of severity and city (*P*=0.000). The frequency of hypotaurodontism, mesotaurodontism and hypertaurodontism was not significantly different between males and females (*P*>0.05). 

Table 2 shows the prevalence of hypotaurodontism, mesotaurodontism and hypertaurodontism in the first and second molars in the right and left quadrants of the maxilla and mandible. The prevalence of taurodontism in general was as follows: maxillary second molars followed by mandibular second molars, maxillary first molars and mandibular first molars, in order of appearance. The prevalence of taurodontism in second molars was more than four times the rate in the first molar teeth and this difference was statistically significant (*P*=0.000). 

In terms of number of taurodont teeth in each individual, one taurodont tooth was noted in 276, two in 119, three in 63, four in 34, five in 13 and six taurodont teeth in eight patients.

**Table1:** The frequency of taurodontism based on gender

		**Male**	**Female**	**Total**
**Taurodont**	**Frequency**	276	266	542
	**Percentage**	22.6	23.3	22.9
**Normal**	**Frequency**	942	876	1818
	**Percentage**	77.4	76.7	77.1
**Total**	**Frequency**	1218	1142	2360
	**Percentage**	100	100	100

**Table 2:** The frequency and percentage of hypotaurodont, mesotaurodontism and hypertaurodontism of first and second molars in the maxilla and mandible

			**Hypotaurodont**	**Mesotaurodont**	**Hypertaurodont**
**First molar**	**Mandible**	**Left**	1.09	0.36	0
		**Right**	0.72	0	0.36
	**Maxilla**	**Left**	8.91	0.45	0
		**Right**	7.64	0.36	0.45
**Second molar**	**Mandible**	**Left**	9.82	3.72	0.63
		**Right**	12.27	3.72	1.45
	**Maxilla**	**Left**	19.18	3.45	0.73
		**Right**	21.27	2.27	1.09

## Discussion

Taurodontism is a dental anomaly, which is seen in both syndromic and non-syndromic normal individuals. It occurs in both maxilla and mandible and is seen in both primary and permanent dentitions. In the current study, the mean prevalence of taurodontism was found to be 22.9%, and the difference in this regard among the eight cities was statistically significant. 

By using biometric method in the mandibular first molars, Seow and Lai [22] showed that the diagnosis of taurodontism was not significantly different on panoramic and parallel periapical radiographs. Many Studies have reported different prevalence rates for taurodontism. For example, this rate was reported to be 8.6% by Ghaznawi *et al.* [15] in Saudi Arabia, 3.9% by Park *et al.* [17] in Korea, 5.5% by Bronoosh *et al*. [18] in the south of Iran, 2.5% by Gupta and Saxena [20] in India, 12% by Munir *et al*. [23] in Pakistan and 48% (18.8% of the teeth) by Sarr *et al.* [24] in Senegal. In the current study, the prevalence of taurodontism was higher in Sari and Karaj and lower in Ahwaz and Zahedan. Different prevalence rates reported in the above-mentioned studies are attributed to racial and ethnic differences, methodology of studies, variable definitions used for detection of taurodontism and different samples (populations or individual teeth). In this study, the prevalence of hypertaurodont and mesotaurodont teeth was 4.8% and 11.07%, respectively. These teeth are detected on the first look on radiographs but detection of hypotaurodont teeth requires accurate measurements. Many studies only performed measurements or calculated the ratios of dental parameters [11, 18, 20, 25]; while in the current study, both methods were used to overcome radiographic shortcomings such as distortion. Previous studies considered teeth with more apical furcation area and larger pulp chambers as taurodont [26, 27] while another study defined taurodont teeth as those with the distance from the furcation area to cementoenamel junction larger than the occluso-cervical height of tooth [28]. In some other studies [14, 29], the criterion for detection of taurodontism was the ratio of total length of tooth to total length of crown. Moreover, prevalence of taurodontism in some previous studies has been calculated as the frequency percentage of patients who have taurodont teeth in the study population while some others calculated the prevalence of taurodontism as the ratio of taurodont teeth to the total number of examined teeth [20, 30-32].

Another reason for variable prevalence rates reported in different studies is the variability of sample size. The smaller sample size, the more unrealistic the prevalence rate. Based on the prevalence of taurodontism in different countries, sample size should not be smaller than 1000 individuals in order to obtain reliable results. 

Controversy exists regarding the role of gender in occurrence of taurodontism [4]. In the current study, the prevalence of taurodontism was not significantly different in males and females. However, in the study by Bronoosh *et al.* [18] the prevalence of taurodontism in molar and premolar teeth was significantly higher in females residing in the south of Iran compared to males. Munir *et al.* [23] however, reported higher prevalence of taurodont teeth in Pakistani females than males, which was in contrast to our finding. Since taurodontism may be related to X chromosome, its higher prevalence in women is somehow expected [33]; this finding has been documented in a Chinese population [34]. But, since in many studies [4, 9, 30], including ours, no significant difference was noted in prevalence of taurodontism between males and females, it appears that some other factors and genes may play a role in this regard. Alternatively, absence of a significant difference in this respect in some studies may be due to the low prevalence of this anomaly in the study population. 

In the current study, the prevalence of taurodontism was 34.1% in the mandible and 65.9% in the maxilla and this difference was statistically significant. This finding is in agreement with previous studies [24, 34]. In contrast, Park *et al*. [17] showed significantly higher prevalence of taurodontism in the mandible compared to the maxilla and according to Burklein *et al.* [30] the occurrence of taurodontism was not significantly different in the maxilla and mandible.

Such variability in the results of the studies may be due to ethnic and racial differences and the sample size. Also, in our study, the prevalence of taurodontism was equal in the right and left quadrants of the jaws, which was similar to the findings of Gupta and Saxena [20] in an Indian population, Bürklein *et al.* [30] in a German population and MacDonald-Jankowski and Li [34] in a Chinese population. Moreover, the prevalence of more severe types of taurodontism namely mesotaurodont and hypertaurodont teeth were lower compared to hypotaurodontism in our study. The same results were obtained by Bronoosh *et al.* [18] and Bürklein *et al*. [30] but Munir *et al*. [23] reported higher frequency of mesotaurodont teeth. This controversy may be attributed to racial differences or the criteria used for assessment of severity of taurodontism. Our results showed significantly higher frequency of taurodontism in the second molars compared to the first molars, which was in agreement with the results of Bronoosh *et al.* [18], Gupta and Saxena [20], Sarr *et al.* [24] and MacDonald-Jankowski and Li [34]. To the best of authors’ knowledge, studies on multiple and/or bilateral taurodontism are scarce and the available ones are mainly case reports [35-37]. Multiple and/or bilateral taurodontism may be associated with some syndromes [38-40]. 

Large sample size and setting strict exclusion criteria were among the strengths of this study and increased the reliability of our findings. Not assessing the prevalence of this anomaly based on the ethnicity of patients was a limitation of this study. Future studies are recommended to assess the prevalence of this dental anomaly in different ethnic groups residing in Iran. Also, considering the probable role of genetic factors, genotyping of patients with taurodontism by use of DNA markers is recommended. 

## Conclusion

Within the limitations of this study, the results showed relatively high prevalence of taurodontism in Iran with no significant difference between males and females. The prevalence of taurodontism was significantly higher in the second molar compared to the first molar teeth and in the maxilla compared to the mandible. In terms of severity, hypotaurodontism was the most common type. Different prevalence rates of taurodontism in different cities of Iran may highlight the role of geographical location and ethnicity in occurrence of this anomaly.
